# Effects of cigarette smoke condensate on proliferation and wound closure of bronchial epithelial cells *in vitro*: role of glutathione

**DOI:** 10.1186/1465-9921-6-140

**Published:** 2005-11-25

**Authors:** Fabrizio Luppi, Jamil Aarbiou, Sandra van Wetering, Irfan Rahman, Willem I de Boer, Klaus F Rabe, Pieter S Hiemstra

**Affiliations:** 1 Department of Pulmonology, Leiden University Medical Center, P.O. Box 9600, 2300RC, Leiden, The Netherlands; 2 Department of Environmental Medicine, Division of Lung Biology and Disease, University of Rochester Medical Center, Rochester, NY 14642, USA

## Abstract

**Background:**

Increased airway epithelial proliferation is frequently observed in smokers. To elucidate the molecular mechanisms leading to these epithelial changes, we studied the effect of cigarette smoke condensate (CSC) on cell proliferation, wound closure and mitogen activated protein kinase (MAPK) activation. We also studied whether modulation of intracellular glutathione/thiol levels could attenuate CSC-induced cell proliferation.

**Methods:**

Cells of the bronchial epithelial cell line NCI-H292 and subcultures of primary bronchial epithelial cells were used for the present study. The effect of CSC on epithelial proliferation was assessed using 5-bromo-2-deoxyuridine (BrdU) incorporation. Modulation of epithelial wound repair was studied by analysis of closure of 3 mm circular scrape wounds during 72 hours of culture. Wound closure was calculated from digital images obtained at 24 h intervals. Activation of mitogen-activated protein kinases was assessed by Western blotting using phospho-specific antibodies.

**Results:**

At low concentrations CSC increased proliferation of NCI-H292 cells, whereas high concentrations were inhibitory as a result of cytotoxicity. Low concentrations of CSC also increased epithelial wound closure of both NCI-H292 and PBEC, whereas at high concentrations closure was inhibited. At low, mitogenic concentrations, CSC caused persistent activation of ERK1/2, a MAPK involved in cell proliferation. Inhibition of cell proliferation by high concentrations of CSC was associated with activation of the pro-apoptotic MAP kinases p38 and JNK. Modulation of intracellular glutathione (GSH)/thiol levels using N-acetyl-L-cysteine, GSH or buthionine sulphoximine (BSO), demonstrated that both the stimulatory and the inhibitory effects of CSC were regulated in part by intracellular GSH levels.

**Conclusion:**

These results indicate that CSC may increase cell proliferation and wound closure dependent on the local concentration of cigarette smoke and the anti-oxidant status. These findings are consistent with increased epithelial proliferation in smokers, and may provide further insight in the development of lung cancer.

## Background

Cigarette smoke, the major risk factor for COPD and lung cancer, contains over 4,500 chemical compounds, including free radicals and oxidants. These compounds are present in both the gas and the tar phase [1] and have been shown to cause epithelial lung injury [2,3]. Epithelial integrity is normally restored by a repair process, that may also result in squamous cell metaplasia and/or goblet cell hyperplasia, especially after repeated injury [4]. This altered composition of the airway epithelium can be observed in smokers [5]. Although these epithelial changes have been observed both for smokers with and without airflow obstruction, some of these epithelial features are more pronounced in COPD patients than in asymptomatic smokers [6,7]. Furthermore, analysis of bronchial biopsies from smokers with chronic bronchitis showed an increased epithelial cell proliferation [8], and studies in both current and former smokers revealed epithelial cell proliferation at sites of metaplasia [9]. These studies indicate that epithelial cell proliferation is a key feature of the epithelial changes observed in smoking-induced lung disease.

Oxidative stress is considered to play a main role in the pathogenesis of inflammatory lung disease, including chronic obstructive pulmonary disease (COPD) [3]. In smokers, this oxidative stress may result both from cigarette smoke itself, and from oxidants released by inflammatory cells that are recruited as a result of smoke-induced injury. The potential importance of oxidative stress in COPD is supported by various studies such as those showing an increase in markers of oxidative stress in patients with COPD [3]. The airway epithelium is a main target for exogenous oxidants such as those present in cigarette smoke. Oxidative stress not only induces cell injury, but also appears to play a central role in e.g. gene expression and cell proliferation. An efficient anti-oxidant system that is present in the lung provides protection against these oxidants, and glutathione (GSH) is considered to be a main antioxidant molecule [10].

Epithelial cell proliferation, as well as various other cellular processes in epithelial cells, is regulated at least in part by Epidermal Growth Factor (EGF)-like factors and the EGF receptor (EGFR). Analysis of the expression of EGF-like growth factors and EGFR in human lung disease has provided evidence for a role of these factors in epithelial remodeling. Kurie *et al*. observed that EGFR expression was increased in metaplastic bronchial epithelium, and reversal of bronchial metaplasia was associated with decreased EGFR expression [11]. Furthermore, Vignola *et al*. observed that EGF expression was significantly increased in chronic bronchitis patients in comparison with healthy non-smokers [12]. Both these studies suggest a role for EGFR and its ligands in the epithelial pathological features observed in smokers with and without COPD.

Downstream signaling pathways that are activated via the EGFR and regulate cell survival and proliferation include phosphorylation of mitogen activated protein kinases (MAPK) and Akt/PI-3 kinase pathways. Activation of the MAPK extracellular-regulated kinase (ERK) 1/2 has been associated with cell survival and proliferation, whereas c-jun N-terminal kinases (JNK) and p38 MAPK are linked to induction of apoptosis [13]. In addition to ligands of the EGFR, oxidants have been shown to cause activation of EGFR [14]. Therefore, oxidants may not only cause direct killing of epithelial cells, but also activate specific signaling pathways. Anti-oxidants such as N-acetyl-L-cysteine (NAC) have been found to be an important tool to study the cellular consequences of oxidative stress. Such studies have shown that the increase in cellular GSH/thiol provided by NAC protects cells against oxidative stress.

The aim of the present study was to analyze the effect of cigarette smoke on cell proliferation and wound repair using an *in vitro *cell culture model. The underlying mechanisms were explored by analyzing the role of MAPK activation and the contribution of an oxidant/antioxidant imbalance in these cellular functions.

## Materials and methods

### Preparation of cigarette smoke condensate (CSC)

Commercial (Caballero, British American Tobacco Group) and standard research cigarettes (Research cigarettes produced for the University of Kentucky Research Foundation, Reference cigarette: code 1R3, date 3/74) were used in this study. CSC was prepared immediately before use essentially as described by Kim JK *et al*. [15]. Briefly, cigarette smoke derived from one cigarette was drawn slowly into a 50 ml glass syringe and bubbled into a tube containing 1 ml of phosphate-buffered saline (PBS), at room temperature. Each cigarette was completely burned after an average of 8 draws of the syringe, with each individual draw taking approximately 10 seconds to complete. The pH of the CSC solution was between pH 7.0 and 7.4. Subsequently, the CSC was filtered through a 0.22 μm pore filter (Schleicher & Schuell GmbH, Dassel, Germany). To prevent possible inactivation of compounds present in the CSC, the CSC was kept in the dark. The concentration of CSC in the solution was calculated by measuring the OD value of the 100-fold diluted solution at a wavelength at which the maximal absorbance (OD_max_) was detected. In the CSC solution this OD_max _was achieved between OD 270–280. The pattern of absorbance observed showed very little difference between different batches of CSC. The concentration, expressed as arbitrary units (AU) per ml, was calculated based on the following formula: OD_max _× 2 × dilution factor. The CSC was further diluted to the required concentration in culture medium. Ten AU/ml was found to correspond to a mean of 5 % (vol/vol) CSC. Bronchial epithelial cells were exposed to various concentrations of CSC within 30 min after CSC preparation.

### Cell culture

NCI-H292 cells, a human pulmonary muco-epidermoid carcinoma cell line, were obtained from the American Type Culture Collection (ATCC, Manassas, VA). The cells were routinely cultured in RPMI 1640 (Gibco, Grand Island, NY) medium containing 2 mM L-glutamine, 20 U/ml penicillin, 20 μg/ml streptomycin (all from Bio Whittaker, Walkersville, MD), and 10% heat-inactivated FCS (Gibco) at 37°C in a humidified 5% CO_2 _atmosphere. Cells were passaged weekly using Trypsin Versene (Bio Whittaker, Walkersville, MD), and starved for growth factors by overnight incubation in serum-free medium before exposure to CSC.

Subcultures from primary bronchial epithelial cells (PBEC) were derived from bronchial tissue that was obtained from resected lungs, derived from patients that underwent lung surgery for lung cancer at the Leiden University Medical Center (Leiden, The Netherlands). In this study, we used cells obtained from seven smokers: four without airflow limitation (FEV_1 _> 81% of the predicted value) and three with airflow limitation (FEV_1 _< 70% of the predicted value). PBEC were isolated from bronchial rings using enzymatic digestion of tissue as previously described [16]. For the experiments, cells from passage two were cultured in DMEM/Ham F12 (1:1) medium (Gibco) supplemented with 10 ng/ml recombinant EGF (Sigma), 2% (v/v) Ultroser G (Gibco), 1 μM isoproterenol, 1 μM insulin (Sigma), 1 μM hydrocortisone (Sigma), 2 mM L-glutamine, 1 mM Hepes (Gibco), 20 U/ml penicillin and 20 μg/ml streptomycin. PBEC were cultured in tissue culture plates precoated with 10 μg/ml fibronectin (isolated from human plasma), 30 μg/ml Vitrogen (Cohesion technologies Inc., Palo Alto, CA) and 10 μg/ml bovine serum albumin (Sigma Chemical Co.).

Prior to the experiments, PBEC were starved for growth factors by overnight incubation in DMEM/HamF12 medium without UltroSer and EGF.

### Cell proliferation

Cell proliferation was assessed using 5-bromo-2-deoxyuridine (BrdU) incorporation as previously described [17]. Briefly, after stimulation, cells were incubated with BrdU (Sigma) for 20 or 24 hours in the presence of the stimulus in starvation medium. Cells were washed twice in PBS and fixed in ethanol 70% (v/v) for at least 1 hour. Cells were then permeabilized with 1 M hydrochloric acid followed by subsequent washes with 0.1 M sodium tetraborate and PBS. BrdU incorporation was demonstrated by incubation with a mouse anti-BrdU mAb followed by incubation with a peroxidase-labeled rabbit anti-mouse polyclonal antibody (both Dako, Glostrup, Denmark). BrdU incorporation was visualized using Nova RED (Vector Laboratories, Burlingame, CA) and the percentage BrdU-positive nuclei was calculated. The percentage BrdU positive nuclei was determined from images that were collected using a digital camera and Axiovision (Carl Zeiss Vision GmbH, München-Hallbermoos, Germany) and Adobe Photoshop (Adobe Systems Incorporated, San Jose, CA) software.

To study the role of oxidants and GSH in the effects of CSC, cells were exposed to N-acetyl cysteine (NAC; Sigma) at 1 mM and CSC. In other experiments, cells were preincubated with DL-buthionine sulphoximine (BSO, Sigma) for 12 hours at a concentration of 10 μM before addition of CSC; BSO was also present during CSC exposure.

### Wound closure model

Epithelial wound closure was studied essentially as described by Aarbiou *et al*. [18]. Both NCI-H292 and PBEC were cultured to confluence. After overnight starvation for growth factors, three circular wounds of 3 mm in diameter were prepared in each well using a Pasteur pipette with sharpened silicone tube. After washing with PBS to eliminate debris, cells were allowed to recover for one hour in starvation medium and subsequently incubated in starvation medium in presence or absence of CSC or TGF-α. In experiments using BSO, cells were pretreated for 12 hours prior to stimulation. NAC was replaced every 12 hours. Images of wounded areas were collected using a digital camera and Axiovision software (Carl Zeiss Vision GmbH, Munchen-Hallbermoos, Germany) at the start of the experiments and at various time points as indicated. Images were used to determine the percentage remaining wound area as compared to the start of the experiment (t = 0) using the Axiovision interactive measurement module (Carl Zeiss Vision).

### Immunoblotting for ERK1/2

PBEC and NCI-H292 cells were cultured to confluence, starved overnight and subsequently stimulated with transforming growth factor (TGF)-α (20 ng/ml) or various concentrations of CSC for 15 minutes, 1, 6 or 24 hours. After washing with washing buffer (5 mM Tris, pH 6.4, 100 mM NaCl, 1 mM CaCl_2_, 1 mM MgCl_2_), cells were lysed in ice-cold lysis buffer (0.5% [v/v] Triton X-100, 0.1 M Tris-HCl pH 7.4, 100 mM NaCl, 1 mM MgCl_2_, 1 mM CaCl_2 _1 mM Na_3_VO_4_, mini complete protease inhibitor cocktail [Roche, Basel, Switzerland]). Following incubation for 10 minutes on ice, cell lysates were centrifuged at 13,000 rpm for 5 minutes at 4°C to remove insoluble debris. Aliquots of the samples containing equal amounts of protein were suspended in reducing SDS-PAGE sample buffer and boiled for 5 minutes. Proteins were separated by 10% SDS-PAGE and transferred to polyvinylidene difluoride (PVDF) membranes using the Mini-transblot system (both Biorad, Hercules, CA). These membranes were incubated in blocking buffer (0.05% Tween-20 in PBS containing 0.5% (w/v) casein) for one hour, followed by overnight incubation with rabbit antibodies directed against total (t) ERK1/2 and phosphorylated (p) ERK1/2 at 4°C (New England Biolabs, Beverly, MA). After incubation with a secondary horseradish peroxidase (HRP) conjugated goat anti-rabbit polyclonal antibody (BD Transduction Laboratories, Franklin Lake, NJ), immunoreactivity was detected by electrochemiluminescent (ECL) detection system (Amersham Pharmacia Biotech, Uppsala, Sweden). In selected experiments, cells were preincubated with the inhibitor of EGFR tyrosine kinase activity AG1478 (Calbiochem, La Jolla, CA).

### Immunoblotting for p38 and JNK

Subconfluent cell cultures were starved overnight and stimulated with TGF-α (20 ng/ml) and various concentrations of CSC for 15 minutes, 1, 6 and 24 hours in RPMI 1640 medium containing glutamine, penicillin and streptomycin. After washing with ice-cold PBS, stimulated cells were lysed with reducing sample buffer and incubated for 10 minutes on ice. Proteins were separated by SDS-PAGE using 10% acrylamide gels and proteins were then transferred to nitrocellulose membrane (Schleicher & Schuell GmbH, Dassel, Germany). These were incubated with 0.05% Tween-20 in Tris Buffered Saline (TBST) containing 5% (w/v) skimmed milk (ELK, Campina, Zoetermeer, The Netherlands) for at least one hour, followed by incubation with antibodies directed against total and phosphorylated p38 and JNK at 4°C (New England Biolabs, Beverly, MA), diluted in TBST. After incubation with horseradish peroxidase (HRP) conjugated donkey anti-rabbit polyclonal antibody (Amersham Pharmacia Biotech, UK), immunoreactivity was visualized as described above.

### Measurement of cellular GSH content

GSH content of epithelial cells was assessed in cellular lysates that were prepared after washing the cells with ice-cold PBS [19]. Briefly, washed cells were lysed by adding ice-cold lysis buffer (0.6 % [w/v] sulfosalicylic acid, 0.1 % [v/v] Triton X-100, 5 mM EDTA in 0.1 M potassium phosphate buffer, pH 7.5) and incubation for 10 min on ice. Lysates were harvested and cell pellets, obtained after centrifugation, were disrupted using a Teflon pestle followed by vortexing. This solution was cleared by centrifugation, and the GSH content of the supernatant was assessed using the method of Tietze [20]. GSH content was calculated using a standard curve, and expressed as nmol/mg protein. The protein content of the lysates was determined using the bicinchonic acid (BCA) method (Pierce Chemical Co, Rockford, IL). In the experiments where the effect of NAC was assessed, cells were preincubated for 16 hours with NAC.

### Statistical analysis

The data are expressed as mean ± SEM. Statistical analysis was performed with Student's t test for paired samples following analysis of variance. Differences were considered statistically significant when p < 0.05.

## Results

### Effect of cigarette smoke condensate (CSC) on cell proliferation

**Figure 1:**
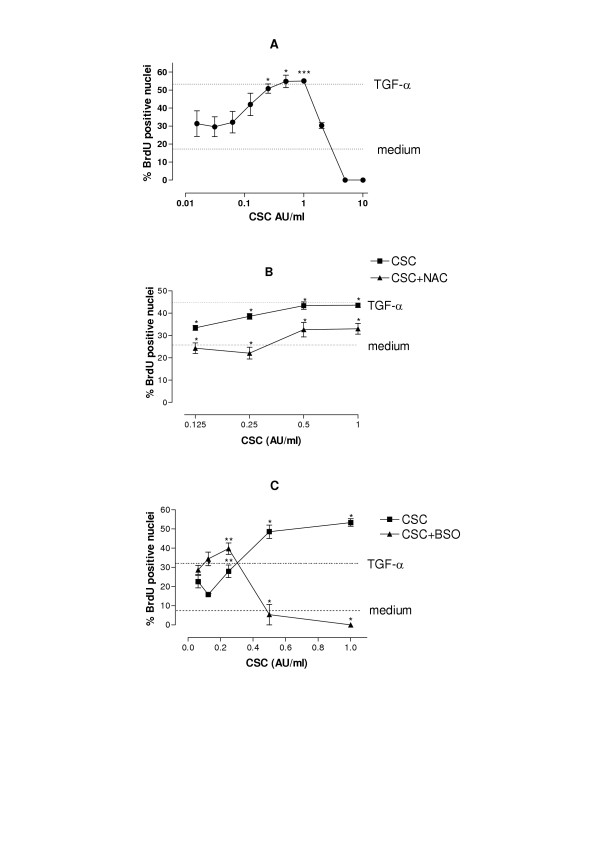
**Effect of CSC and its modulation by BSO and NAC on cell proliferation in NCI-H292. **Subconfluent cultures of NCI-H292 cells were incubated for 24 h with various concentrations of CSC (A), CSC and NAC (B) or preincubated for 16 h with BSO (10 μM), followed by the addition of freshly prepared CSC (C). Next, BrdU was added and the cells were incubated for another 4 h and subsequently washed and fixed. BrdU incorporation was detected by immunocytochemistry (for details see material and methods). Horizontal bars indicate BrdU incorporation observed with TGF-α or medium alone. The results are mean ± SEM of 3 independent experiments, each performed in duplicate. Note the difference in scaling of the x-axes. * p < 0.05; ** p < 0.001; *** p < 0.001 vs medium-treated cells (Fig. 1A) or vs cells exposed to the same concentration of CSC in absence of NAC (Fig. 1B) or BSO (Fig. 1C).

The effect of CSC on cell proliferation was studied using BrdU incorporation in subconfluent cultures of NCI-H292 bronchial epithelial cells. CSC caused a dose-dependent increase in cell proliferation at low concentrations (0.25 – 1 AU/ml) after 24 hours, whereas higher concentrations decreased cell proliferation (Fig. 1A). Comparable results were obtained using the tetrazolium salt MTT [3-(4, 5-dimethylthiazol-2-yl)-2, 5-diphenyltetrazolium bromide] assay to assess viable cells by determining mitochondrial activity (data not shown). Preincubation of the cells with the antioxidant NAC (1 mM) markedly reduced the mitogenic effect of low concentrations of CSC (Fig 1B), whereas it partially restored cell proliferation in the presence of 5 or 10 AU/ml CSC (data not shown). In line with this finding, NAC also prevented CSC-induced cytotoxicity, as demonstrated by trypan blue exclusion (data not shown). In contrast, when intracellular GSH was depleted using buthionine sulphoximine (BSO; 10 μM), an inhibitor of glutamate cysteine ligase, GCL (formerly known as γ-glutamylcysteine synthetase, γ-GCS), cell proliferation at mitogenic concentrations of CSC (1 and 0.5 AU/ml) was markedly reduced, whereas proliferation in cells incubated with submitogenic concentrations of CSC (0.125 and 0.25 AU/ml) was increased (Figure 1C). These results demonstrate that modulation of intracellular GSH affects both the mitogenic and the toxic effects of CSC.

### Effect of CSC on epithelial wound repair

Since epithelial cell proliferation plays a central role in epithelial wound closure, we next assessed whether the effects of CSC on epithelial cell proliferation in subconfluent cultures were also reflected by similar findings in a model of epithelial wound closure. Therefore we used a model that we recently developed [18], in which closure of wounds prepared by scraping a defined circular wound in a confluent layer of NCI-H292 cells or PBEC is studied.

**Figure 2:**
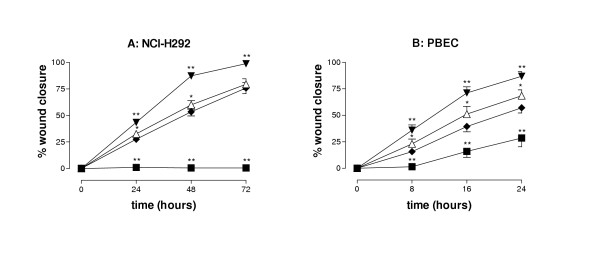
**Effect of CSC on epithelial wound closure in NCI-H292 cells and PBEC**. Mechanical wounds were prepared in monolayers of NCI-H292 (panel A) or PBEC (panel B) and the area of the wound was determined at various time points as indicated and used to calculate the % wound closure. Following wounding, the cultures were incubated with freshly prepared CSC (5 or 1 AU/ml; ■, △), TGF-α (▼; 20 ng/ml) or medium alone (◆). The results in panel A (NCI-H292 cells) are mean ± SEM of 6 independent experiments, each performed in triplicate. The results in panel B (PBEC) are mean ± SEM of PBEC cultures derived from 7 different donors. ** P < 0.004; * P < 0.05 vs medium alone

In NCI-H292 cells, TGF-α caused a marked increase in wound closure at all time points studied (Figure 2A). At 5 AU/ml, CSC completely inhibited wound closure as a result of cytotoxicity (demonstrated using trypan blue exclusion; data not shown). In contrast, CSC at 1 AU/ml caused a limited, but significant (p = 0.05, at 24 and 48 h) increase in wound closure. Essentially similar results were obtained when studying wound closure in PBEC (Figure 2B), although wounds prepared in PBEC cultures closed faster. Also in PBEC, TGF-α increased wound closure at all time points studied. Whereas at 5 AU/ml, CSC inhibited wound closure (p < 0.002 vs. medium control), at 1 AU/ml a significant increase in wound closure was observed at all time points (p < 0.02 vs. medium control) (Figure 2B).

**Figure 3:**
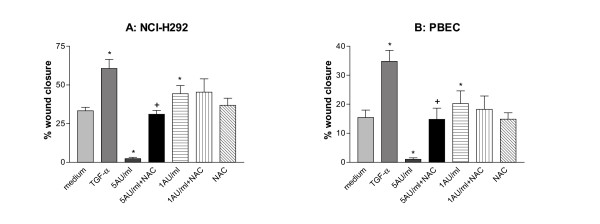
**Effect of N-acetylcysteine (NAC) on CSC-induced epithelial wound repair in NCI-H292 and in PBEC. **Mechanical wounds prepared in monolayers of NCI-H292 (panel A) or PBEC (panel B). Following wounding, the cultures were incubated for 24 h (NCI-H292; panel A) or 8 h (PBEC; panel B) with freshly prepared CSC (5 or 1 AU/ml), TGF-α (20 ng/ml), medium alone, NAC (1 mM) (alone or in combination with CSC). Next the residual wound area was determined and used to calculate the % wound repair. Similar results were obtained when analyzing wound closure at 72 h (data not shown). The results in NCI-H292 cells are mean ± SEM of 3 independent experiments, each performed in triplicate. The results in panel B are mean ± SEM of PBEC cultures derived from 5 different donors. The cultures from the different donors were performed on different days, and each experiment was performed in triplicate. * p < 0.05 vs. medium alone; + p < 0.05 vs. CSC 5 AU/ml

**Figure 4:**
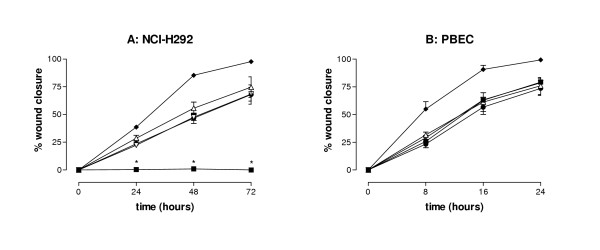
**Effect of buthionine sulphoximine (BSO) on CSC-induced epithelial wound closure in NCI-H292 and in PBEC. **Mechanical wounds prepared in monolayers of NCI-H292 (panel A) or PBEC (panel B). Before wounding, the cultures were preincubated for 16 hours with BSO, followed by incubation for 72 h (NCI-H292; panel A) or 24 h (PBEC; panel B) with freshly prepared CSC (△; 1 AU/ml), TGF-α (◆; 20 ng/ml), medium alone (●), BSO (alone [▽] or in combination with 1 AU/ml of CSC [■]). Next the residual wound area was determined and used to calculate the % wound repair. The results of both the experiments are mean ± SEM of 3 independent experiments, each performed in triplicate. P < 0.03 vs CSC 1 AU/ml.

Whereas NAC significantly restored wound closure in both NCI-H292 and PBEC treated with 5 AU/ml CSC, it did not affect wound closure in presence of 1 AU/ml (Figure 3). The same results were obtained after incubating cells with GSH (1.25 – 5 mM) instead of NAC (data not shown). In contrast, in NCI-H292 cells but not in PBEC, depleting GSH using BSO resulted in a full inhibition of wound repair in presence of 1 AU/ml that was accompanied by cytotoxicity (Figure 4). Also higher concentrations of BSO (150 μM) did not affect wound closure in presence of 1 AU/ml CSC (data not shown). These results demonstrate the crucial involvement of oxidants/free radicals in the inhibitory effects of CSC on wound closure, and illustrate the differential sensitivity of NCI-H292 and PBEC to oxidative stress induced by CSC.

When studying epithelial wound repair, essentially no difference was observed between the effect of CSC prepared from commercial brand cigarettes and that from University of Kentucky standard research cigarettes (data not shown). Therefore, all experiments were performed using CSC prepared from commercial brand cigarettes.

### Effect of CSC on cell proliferation during epithelial wound closure

The stimulatory effects of CSC on wound closure were less pronounced than their effect on cell proliferation in subconfluent cultures. Since epithelial wound closure is mediated by both cell migration and proliferation, and since it has been described that CSC inhibits epithelial migration [21], we next investigated the effect of CSC on cell proliferation using BrdU incorporation in cells present in the original wound area at different phases of the repair process.

The results revealed that CSC had similar effects on cell proliferation in the original wound area in the wound closure model in NCI-H292 (Table 1), as observed with subconfluent cultures of NCI-H292 (Figure 1). First, the percentage of BrdU positive cells was higher in the wound area compared to cells outside the wound area, irrespective of the conditions tested. Second, TGF-α and 1 AU/ml CSC caused an increase in BrdU incorporation in cells present within and outside the original wound area already after 24 hours (Table 1). No BrdU incorporation was observed in cultures incubated with 5 AU/ml.

**Table 1:** Cell proliferation in mechanically wounded NCI-H292 cell monolayers: effect of CSC.

	% BrdU positive nuclei^a^			
Stimuli	BrdU incubation period			
	24–48 h		48–72 h	

	wound	intact	wound	intact
	
Medium	21.5 ± 0.7	17.0 ± 1.6	20.9 ± 1.0	12.4 ± 1.4
TGFα	47.3 ± 3.9*	34.9 ± 1.5*	27.5 ± 0.5*	16.4 ± 2.2
NAC	22.5 ± 2.9	16.3 ± 1.8	20.5 ± 1.1	14.4 ± 1.3
CSC 1 AU/ml	39.8 ± 1.0*	29.5 ± 3.8*	42.2 ± 1.6*	24.0 ± 2.3
CSC 1 AU/ml+NAC	25.7 ± 0.7ˆ	17.4 ± 2.7ˆ	21.8 ± 2.5ˆ	16.0 ± 1.8ˆ

^a^Percentage BrdU-positive nuclei within a distance of 10 cells from the wound edge (i.e. within the original wound area) and in intact areas. Data are mean ± SEM of 3 independent experiments. *: p < 0.05 versus serum-free medium-treated cells. ˆ:p < 0.05 vs CSC 1 AU/ml

In wounded PBEC layers, the percentage of proliferating cells was lower (< 10 % BrdU positive nuclei) than observed in wounded NCI-H292 monolayers. Within one well, the percentage of proliferating PBEC was lower inside the original wound area as compared to outside this area (data not shown). As it appears that cell proliferation does not markedly contribute to wound closure in the PBEC model, the effect of CSC on proliferation in this model was not further explored.

In summary, these data indicate that CSC has dual effects on epithelial cell proliferation by increasing proliferation at low, and decreasing proliferation at high concentrations both in subconfluent layers of epithelial cells and during wound closure.

### Effect of CSC on epithelial GSH content

**Figure 5:**
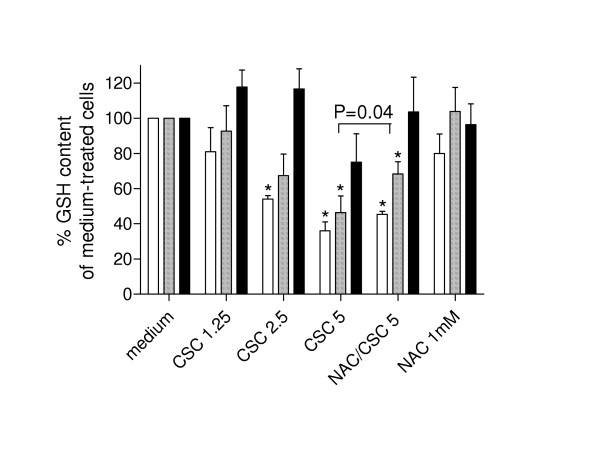
**Intracellular GSH in NCI-H292 after exposure to CSC. **NCI-H292 cells were exposed to various concentrations of CSC (5, 2.5 and 1.25 AU/ml) in presence or absence of NAC (1 mM), or NAC alone for 1 (open bars), 3 (hatched bars) or 5 h (filled bars). Intracellular GSH content was measured in cellular lysates and expressed as mean percentage ± SEM of that of medium-treated cells. p < 0.05 vs medium-treated cells; GSH content of cells exposed to 5 AU/ml CSC alone or with NAC differed significantly at 3 h (P = 0.04).

To investigate the effect of CSC on intracellular GSH in the epithelial cells used, the intracellular GSH content of NCI-H292 cells was assessed at different times after exposure to CSC. The results show that CSC causes a time and dose-dependent decrease in GSH, that was partly prevented by preincubation with NAC (Figure 5).

### Effect of CSC on MAPK activation

**Figure 6:**
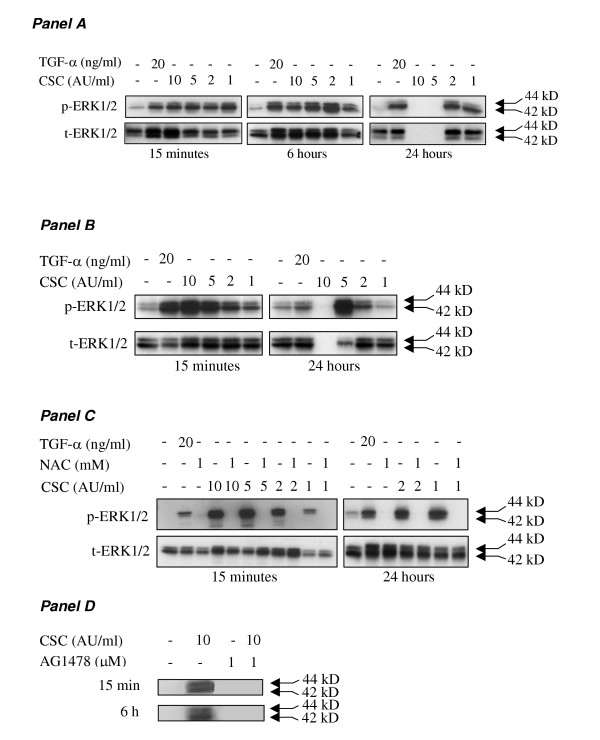
**CSC-induced phosphorylation of Extracellular signal Regulated Kinases (ERK1/2) 1/2. **NCI-H292 cells (A and C) were stimulated with medium, TGF-α, and CSC (10, 5, 2 and 1 AU/ml) for 15 minutes, 6 and 24 hours (A) or with medium, TGF-α, and 2 and 1 AU/ml of CSC in presence (C) or absence (A) of NAC. PBEC were stimulated with medium, TGF-α and CSC (10, 5, 2 and 1 AU/ml) for 15 minutes and 24 hours (B). Next cellular lysates were prepared, and used to detect the level of total (t-ERK1/2) and phosphorylated ERK1/2 (p-ERK1/2) using Western blot analysis. The results shown in each panel are from one experiment that was repeated three times with similar results. To assess the role of EGFR in CSC-induced ERK1/2 phosphorylation, NCI-H292 cells were preincubated for 1 hour with 1 μM AG1478, and incubated for 15 minutes or 6 hours with medium alone or with CSC (10 AU/ml) (D).

**Figure 7:**
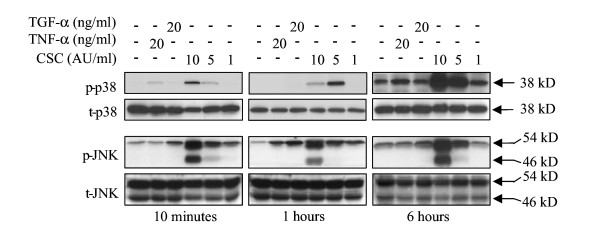
**CSC-induced phosphorylation of p38 and JNK. **NCI-H292 cells were stimulated with medium, TGF-α, TNF-α or CSC (10, 5 and 1 AU/ml) for 10 minutes, 1 and 6 hours. Next cellular lysates were prepared and used to detect the level of total (t-p38 and t-JNK) and phosphorylated (p-38 and t-JNK) p38 and JNK using Western blot analysis. The results shown in each panel are from one experiment that was repeated three times with similar results.

Because various members of the MAPK family play different roles in the regulation of cell fate, the effect of CSC on activation of the MAPK ERK1/2, p38 and JNK was explored (Figure 6 and 7 and data summary in table 2). In these experiments, TNF-α was included as a positive control for activation of p38 and JNK. Whereas ERK1/2 activation was observed at all concentrations of CSC both in NCI-H292 (Figure 6A) and PBEC (Figure 6B), activation of p38 and JNK was only observed at the higher concentrations (5 and 10 AU/ml). Furthermore, ERK1/2 activation was already observed at 15 min and persisted for 24 hours, while p38 and JNK activation was maximal at 6 h; no activation was observed after 24 hours (Figure 7). These results indicate that CSC exerts a concentration- and time-dependent effect on the ratio between activated ERK1/2 and p38/JNK, suggesting a shift towards a predominance of p38/JNK activation at higher CSC concentrations following prolonged incubation. In addition, the persistent activation of ERK1/2 in the absence of notable p38/JNK activation observed with 1 AU/ml, is in line with the proposed role of this MAP kinase in cell proliferation. In the presence of NAC, CSC did not induce ERK1/2 activation, indicating that this process involves the action of oxidants/free radicals (Figure 6C). Our observation that the EGFR tyrosine kinase inhibitor AG1478 blocks CSC-induced phosphorylation of ERK1/2 is in line with studies showing a role of EGFR in cigarette smoke induced epithelial cell activation (Figure 6D).

**Table 2:** Summary of the data on analysis of MAPK ERK1/2, p38 and JNK in NCI-H292 cells following exposure to CSC.

		**Treatment^b^**					
		
**MAPK**	**Time^a^**	medium	TGF-α	TNF-α	CSC (AU/ml)		
					
					10	5	1
ERK1/2	15 min	-	+	±	+	+	+
	1 hour	-	+	±	+	+	+
	6 hours	-	+	-	+	+	+
	24 hours	-	+	-	-	-	+

p38	10 min	-	-	+	+	+	-
	1 hour	-	-	±	+	+	-
	6 hours	-	-	-	++	++	-
	24 hours	-	-	-	-	-	-

JNK	10 min	-	-	-	-	-	-
	1 hour	-	-	-	+	±	-
	6 hours	-	-	-	++	+	-
	24 hours	-	-	-	-	-	-

^a ^Cells were exposed to the various treatments for the indicated time periods. ^b ^The degree of phosphorylation of the three MAPK studied is indicated by the symbol.

## Discussion

The results from the present study show a dose-dependent and dual effect of cigarette smoke on bronchial epithelial cell proliferation and wound repair. In cultures of the bronchial epithelial cell line NCI-H292, proliferation was inhibited at high and stimulated at low concentrations of CSC. Similar effects of CSC on epithelial wound closure in NCI-H292 or PBEC supported these results. Experiments using NAC, GSH and BSO to modify the intracellular thiol status, revealed a critical role of oxidants/free radicals in mediating these effects of CSC. Activation of ERK1/2, a MAPK involved in cell proliferation and survival, was increased by various concentrations of CSC, and sustained up to 24 h only at mitogenic concentrations of CSC (1 AU/ml). Higher, cytotoxic concentrations of CSC resulted in activation of the pro-apoptotic MAPK p38 and JNK. These results suggest an involvement of different MAP kinases in CSC-induced cell proliferation and cytotoxicity.

Various studies have demonstrated marked effects of cigarette smoke and its aqueous extracts on epithelial cell behavior, including proliferation and wound repair. However, most of these studies focused on high, cytotoxic concentrations that were found to inhibit proliferation and wound repair in bronchial epithelial cells [22]. In contrast to our observations, Lannan *et al*. did not observe any increase in proliferation of alveolar A549 epithelial lung adenocarcinoma cells by CSC using 1–10% of CSC [2], which may be a specific feature of these cells or the CSC concentration used. Our results are however in line with the observation that short term exposure of rats to cigarette smoke condensate results in an increase in cell proliferation in the bronchiolar epithelium and the pulmonary vasculature [23]. Furthermore, our conclusions are also supported by studies showing that a broad range of oxidants, other than those present in cigarette smoke, can stimulate epithelial cell proliferation [24-26].

The importance of an oxidant/antioxidant imbalance in regulating both CSC-induced cell death, inhibition of wound repair and mitogenesis was demonstrated in studies using NAC, GSH and BSO. Previous to our study, the importance of GSH in cellular defense against CSC was demonstrated by the ability of both GSH and NAC to protect cells from CSC-induced cell death [2,27]. The inhibitory action of NAC on the effects of CSC may have been the result of both the extracellular scavenging action of NAC or its ability to increase cellular GSH since NAC was present in the culture medium during exposure to CSC. NAC prevented the CSC-induced decrease in GSH, but did not increase GSH in the absence of CSC. This finding is in line with previous observations, showing that NAC does increase total non-protein thiols but does not increase GSH [28,29]. More direct evidence for the involvement of cellular GSH came from our observation that pharmacological inhibition of GCL by BSO increases the sensitivity of epithelial cells to oxidative stress resulting from CSC exposure. This observed effect of BSO may also be relevant for our insight into the way TGFβ expression may alter the response to CSC in the (susceptible) smoker. TGFβ is a pleiotropic cytokine and its expression is higher in smokers with COPD when compared to those without COPD [30]. For the present study it is interesting to note that TGFβ blocks GCL synthesis in cultured epithelial cells [31], and thereby – like BSO, a chemical inhibitor of GCL – may decrease intracellular GSH levels. It needs to be noted that GSH and glutathione *S*-transferases (GSTs) not only protect cells from the action of oxidants, but also play a more general role in the detoxification of electrophilic components that are present in CSC [32]. Therefore the observed depletion of GSH and modulatory effects of NAC and BSO do not provide definitive proof for an exclusive role of oxidants in the observed effects of CSC. GSH can also directly scavenge the electrophilic compounds present in CSC [19].

What is the mechanism involved in the increase in epithelial wound repair and the proliferative response following exposure to low concentrations of CSC? It is known that oxidants and CSC are able to cause ligand independent transactivation of the epidermal growth factor receptor (EGFR) [14]. Reactive oxygen species may employ EGFR phosphorylation to activate MAPK such as extracellular-signal regulated kinase (ERK) 1/2, that in turn may induce shedding of the EGF receptor ligands such as TGFα and thereby lead to further activation of the EGFR [33]. Our finding that the EGFR tyrosine kinase inhibitor AG1478 blocks CSC-induced ERK1/2 activation is in accordance with these observations. In line with this, reports demonstrated the involvement of ADAM 17 (tumor necrosis factor α converting enzyme; TACE) in shedding of TGFα and amphiregulin from bronchial epithelial cells exposed to suspended smoke particles [34,35]. The present study confirms the stimulatory effect of CSC on epithelial cell proliferation, and links this effect to an imbalance between oxidants and antioxidants and to MAPK activation. Our observation that CSC at mitogenic concentrations induces phosphorylation of ERK1/2 suggests a potential role of this MAPK pathway in the development of both epithelial hyperplasia and metaplasia in smokers, features that may predispose to the development of lung cancer [36,37]. Furthermore, Richter *et al*. demonstrated that CSC not only induces release but also increases expression of selected EGFR ligands from airway epithelial cells [38]. Taken together these data and our observation on prolonged activation of ERK1/2 following exposure to subtoxic concentrations of CSC may provide a mechanistic basis for the observed stimulatory effect of CSC on cell proliferation and epithelial wound repair *in vitro*. Future studies are needed to define a functional involvement of ERK1/2 activation in the observed effects of CSC on proliferation and wound closure. Our results are in agreement with a recent study showing that two compounds of cigarette smoke, nicotine and 4-(methylnitrosamino)-1-(3-pyridyl)-1-butanone, activate the serine/threonine kinase Akt leading to increased cell survival and tumorigenesis in human airway epithelial cells [39], and a study showing that nicotine induces cell proliferation in neoplastic epithelial cells [40]. The relative contribution of 4-(methylnitrosamino)-1-(3-pyridyl)-1-butanone and nicotine or other reactive aldehydes to the effects of CSC observed in the present study is not known, also because it is not clear whether intracellular thiols block the stimulatory effect of these components. Furthermore, we have not explored the role of the serine/threonine kinase Akt in the mitogenic effects of CSC observed in the present study. In addition to the MAPK pathway, the Akt/PI-3 kinase pathway may play an important role in CSC-induced epithelial cell proliferation. Finally, a stimulatory effect of aged suspended smoke particles on cultured human bronchial epithelial cells was recently described [34].

At higher concentrations, CSC has been shown to cause cell death, which seems to be due to high concentrations of oxidants and other radicals [27] and this study). Moderate oxidative stress induces apoptosis, whereas necrosis occurs when cells are exposed to a higher dose of oxidants [41,42]. It has been demonstrated that p38 and JNK are involved in oxidant-induced cell death [43], and cell fate is regulated by a balance between all three MAP kinase pathways [44]. Interestingly, at the higher concentrations tested (10 and 5 AU/ml) we observed activation of both pro-apoptotic (p38 and JNK) and pro-survival/proliferation (ERK1/2) MAPK pathways. Based on the observation that these high concentrations of CSC induce cell death, it appears that activation of pro-apoptotic signals predominates. Studies using inhibition of these separate MAPK signaling pathways are needed to delineate their role in the cellular effects of CSC observed in the present study.

In our epithelial wound repair model, we observed differences between NCI-H292 cells and PBEC that are relevant to the interpretation of the effects of CSC on repair. Following injury in NCI-H292 cells, we observed marked proliferation in the cells that covered the original wound area, indicating a contribution of proliferation to the repair process. In contrast, in PBEC cultures very few proliferating cells were present in the wound area, suggesting that in PBEC repair of wounds of the size used in the present study is mainly mediated by migration or cell spreading. Therefore, a stimulatory effect of CSC on proliferation of PBEC could not be observed. Nevertheless, a small but significant effect of low concentrations of CSC on wound closure of both NCI-H292 and PBEC was observed. This may indicate an effect of CSC on cell migration that is dependent on the concentration of CSC used and the cell type investigated. Previously, an inhibitory effect of toxic concentrations of CSC on epithelial migration was reported [21]. We also observed that PBEC appear more resistant to the cytotoxic effects of CSC than NCI-H292. Because of the role of intracellular GSH in mediating cellular defense against oxidative stress resulting from CSC exposure, we hypothesized that GSH levels may differ between PBEC and NCI-H292. Furthermore, since all PBEC cultures used in the present study were derived from smokers, the possibility that bronchial epithelial cells from smokers may display increased GSH levels needs to be considered [3]. In addition, further studies are required to delineate differences between the effects of CSC on NCI-H292 and PBEC. In our study, we have used an aqueous extract of cigarette smoke to gain insight into the effect of cigarette smoke on epithelial cells. Much of our knowledge on the cellular effects of smoke is based on studies using such smoke extracts instead of smoke. Notwithstanding the inherent limitations of this model, it can be argued that epithelial cells in the lung – like those in our cultures – are also exposed to smoke components that have been extracted into a fluid, *i.e*. the epithelial lining fluid [45]. The use of the terms "low" and "high" to describe the CSC concentration does not imply any comparison with actual levels of cigarette smoke compounds in lungs. Since the pulmonary levels of individual compounds of cigarette smoke are unknown, comparisons between the *in vivo *and *in vitro *situation are difficult to make. However, the CSC concentrations used in this study are comparable to the concentrations used in other *in vitro *studies.

In our study we have focussed on the effects of cigarette smoke on proliferation and wound closure in cultures of bronchial epithelial cells. These *in vitro *results may add new elements to our insight into the pathogenesis of smoking-induced lung injury, and more specifically to the epithelial changes that may accompany COPD and chronic bronchitis. Our observation on CSC-induced epithelial cell proliferation suggests that cigarette smoke alone may partly explain the increased amount of proliferating cells observed in bronchial biopsies obtained from smokers [8], and may be relevant for our understanding of mechanisms involved in the epithelial hyperplasia and metaplasia that is frequently observed in smokers with and without airflow limitation [7]. Our findings may also be relevant for our insight in the development of smoking-induced lung cancer, since epithelial metaplasia and hyperplasia induced by cigarette smoke is considered a precancerous lesion [46]. In this respect it is interesting to note that constitutive activation of ERK1/2 may suffice to cause transformation [47], and that carcinoma cells often demonstrate high basal levels of ERK1/2 activation [48]. Therefore, a better understanding of the mechanisms by which cigarette smoke in particular by redox signaling affects wound repair may lead to improved therapeutic interventions for the prevention and treatment of smoking-induced lung disease.

## Conclusion

Our study revealed dual effects of cigarette smoke on epithelial cell behavior. Our observation that low concentrations of CSC induce cell proliferation may be relevant for our understanding of epithelial changes in chronic bronchitis and COPD. Furthermore, our observation that different patterns of MAPK activation are associated with CSC-induced proliferation and cell death provides insight into the cellular response to cigarette smoke, and its putative modulation using pharmacological inhibition of the different MAPK pathways.

## Competing interests

The authors declare that they have no competing interests.

## Authors' contributions

FL carried out the cell culture experiments, analysis of MAP kinase activation and wrote the manuscript. JA, SW and IR introduced techniques used in the present study. WIB, KFR and PSH were involved in the design, supervision and writing of the manuscript. All authors have participated in the study design and evaluation, and have read, contributed and approved the manuscript.
